# Functional and structural connectivity of frontostriatal circuitry in Autism Spectrum Disorder

**DOI:** 10.3389/fnhum.2013.00430

**Published:** 2013-08-06

**Authors:** Sonja Delmonte, Louise Gallagher, Erik O'Hanlon, Jane McGrath, Joshua H. Balsters

**Affiliations:** ^1^Department of Psychiatry, Trinity College DublinDublin, Ireland; ^2^Trinity College Institute of Neuroscience, Trinity College DublinDublin, Ireland; ^3^Department of Psychiatry, Royal College of SurgeonsDublin, Ireland; ^4^Neural Control of Movement Lab, Department of Health Sciences and Technology, ETH ZurichZurich, Switzerland

**Keywords:** Autism Spectrum Disorder, connectivity, frontostriatal, striatum, fMRI, DTI, social reward

## Abstract

Abnormalities in frontostriatal circuitry potentially underlie the two core deficits in Autism Spectrum Disorder (ASD); social interaction and communication difficulties and restricted interests and repetitive behaviors. Whilst a few studies have examined connectivity within this circuitry in ASD, no previous study has examined both functional and structural connectivity within the same population. The present study provides the first exploration of both functional and structural frontostriatal connectivity in ASD. Twenty-eight right-handed Caucasian male ASD (17.28 ± 3.57 years) and 27 right-handed male, age and IQ matched controls (17.15 ± 3.64 years) took part in the study. Resting state functional connectivity was carried out on 21 ASD and control participants, and tractography was carried out on 22 ASD and 24 control participants, after excluding subjects for excessive motion and poor data quality. Functional connectivity analysis was carried out between the frontal cortex and striatum after which tractography was performed between regions that showed significant group differences in functional connectivity. The ASD group showed increased functional connectivity between regions in the frontal cortex [anterior cingulate cortex (ACC), middle frontal gyrus (MFG), paracingulate gyrus (Pcg) and orbitofrontal cortex (OFC)], and striatum [nucleus accumbens (NAcc) and caudate]. Increased functional connectivity between ACC and caudate was associated with deactivation to social rewards in the caudate, as previously reported in the same participants. Greater connectivity between the right MFG and caudate was associated with higher restricted interests and repetitive behaviors and connectivity between the bilateral Pcg and NAcc, and the right OFC and NAcc, was negatively associated with social and communicative deficits. Although tracts were reliably constructed for each subject, there were no group differences in structural connectivity. Results are in keeping with previously reported increased corticostriatal functional connectivity in ASD.

## Introduction

Frontostriatal circuitry plays an important role in social motivation, which is postulated to underlie deficits in social interaction and communication in Autism Spectrum Disorder (ASD) (Dawson et al., 2005, 2012; Chevallier et al., 2012). Aberrant BOLD responses to social rewards have been reported in a number of studies of social reward processing in ASD, providing support for this hypothesis (Scott-Van Zeeland et al., 2010; Dichter et al., 2011; Delmonte et al., 2012; Kohls et al., 2012a,b). Studies of reward and executive function also implicate frontostriatal circuitry in repetitive behavior symptoms (Langen et al., 2011a,b; Dichter et al., 2012). Additionally, functional abnormalities in frontostriatal circuitry have been reported during higher-order cognitive and sensorimotor tasks (Schmitz et al., 2006; Takarae et al., 2007; Scott-Van Zeeland et al., 2010). Therefore, abnormalities in frontostriatal circuitry may underlie the two core deficits in ASD; social interaction and communication, and restricted interests and repetitive behaviors (Langen et al., 2011a,b; Chevallier et al., 2012; Dichter et al., 2012), as well as other cognitive and motor impairments that are associated with ASD.

Frontostriatal circuitry plays a key role in a number of different processes such as emotion, motivation, cognition, and the control of movement, which work in tandem to execute goal directed behaviors (Haber, 2003). The functional variety of frontostriatal circuits can be explained to some extent by examining its cortical inputs. Frontostriatal circuits have a looped structure with cortical inputs feeding information to the striatum which in turn projects back to the cortex via the thalamus (Alexander et al., 1986, 1990). Primate studies have shown that frontostriatal projections are arranged into a number of parallel, integrative loops, with each loop comprising discrete regions of striatum, cortex, globus pallidus, substantia nigra and thalamus, and subserving specific motor, cognitive, or affective function (Groenewegen et al., 1999, 2003; Haber and Knutson, 2009). Information is primarily channelled from ventral limbic, to more dorsal cognitive and motor loops such that action decision-making is influenced by motivation and cognition (Middleton and Strick, 2000; Haber, 2003). Diffusion tensor imaging (DTI) studies indicate that corticostriatal circuitry is similarly organized into segregated and converging loops in humans (Lehéricy et al., 2004; Leh et al., 2007; Draganski et al., 2008; Verstynen et al., 2012) and resting state functional connectivity analysis of the human striatum has shown functional organization of corticostriatal loops into affective, cognitive, and motor components (Di Martino et al., 2008; Choi et al., 2012).

ASD is characterized by abnormal functional and structural connectivity (Just et al., 2004; Cherkassky et al., 2006; Alexander et al., 2007; Keller et al., 2007; Kleinhans et al., 2008; Di Martino et al., 2010; Weng et al., 2010; Langen et al., 2011a,b; Müller et al., 2011; Sato et al., 2012; Von dem Hagen et al., 2012). Despite the growing evidence implicating frontostriatal circuitry in ASD pathology, few studies have specifically examined connectivity within this circuit. In a resting state study of corticostriatal connectivity, children with ASD showed increased connectivity between the caudate and putamen and a number of cortical and subcortical regions (Di Martino et al., 2010). Only one previous DTI tractography study has examined frontostriatal structural connectivity in ASD. The ASD group showed lower fractional anisotropy (FA) in tracts connecting the putamen to the frontal cortex, and increased mean diffusivity (MD) in tracts connecting the NAcc to the frontal cortex (Langen et al., 2011a,b).

To date, no previous study has combined functional and structural MRI data from the same participants to examine the connectivity of frontostriatal circuitry in ASD. In the present study, we investigated functional connectivity between frontostriatal regions and potential white matter differences underlying group differences in functional connectivity. Group differences in connectivity were examined in relation to behavioral impairments and striatal deactivation to social rewards as previously reported in the same particpants (Delmonte et al., 2012).

## Methods

### Participants

Twenty-eight right-handed Caucasian male ASD [mean age (SD) = 17.28 (3.57) years] and 27 right-handed male, age and IQ matched controls [mean age (SD) = 17.15 (3.64) years] took part in the MRI study. Twenty-one ASD and control participants were retained for the fMRI analysis and 22 ASD and 24 control participants were included in the DTI analysis after excluding subjects for excessive motion (movements >3 mm) or poor data quality. ASD participants were recruited through an associated genetics research programme, clinical services, schools and advocacy groups. Controls were recruited through schools, the university and volunteer websites. Ethical approval was obtained from the St. James's Hospital/AMNCH (ref: 2010/09/07) and the Linn Dara CAMHS Ethics Committees (ref: 2010/12/07). Written informed consents/assents were obtained from all participants and their parents (where under 18 years of age).

Exclusion criteria included a Full Scale IQ (FSIQ) <70, known psychiatric, neurological, or genetic disorders, a history of a loss of consciousness for more than 5 min and those currently taking psychoactive medication. Four subjects in the ASD group had a secondary diagnosis of Attention Deficit Disorder (ADD) or Attention Deficit Hyperactivity Disorder (ADHD). Controls were excluded if they had a first degree relative with ASD or scored above 50 on the Social Responsiveness Scale (SRS) (Constantino et al., 2003) or above 10 on the Social Communication Questionnaire (SCQ) (Rutter et al., 2003). The Adult prepublication version of the SRS was used with permission in cases 18 years or older (Constantino and Todd, 2005). All participants had normal, or corrected to normal, vision.

### Diagnostic assessments and cognitive measures

ASD diagnosis was confirmed using the Autism Diagnostic Observation Schedule (ADOS) (Lord et al., 1994) and the Autism Diagnostic Interview Revised (ADI-R; Lord et al., 2000). Clinical consensus diagnosis was established using DSM-IV-TR criteria and expert clinician (Louise Gallagher). FSIQ was measured using the four subtest version of the Wechsler Abbreviated Scale of Intelligence (WASI; Wechsler, 1999) or the Wechsler Intelligence scale for Children-Fourth Edition (WISC-IV; Wechsler, 2003). Performance IQ (PIQ) score was based on the Matrix Reasoning and Block Design subtests and Verbal IQ (VIQ) score on the Vocabulary and Similarities subtests.

### MRI data acquisition

A high-resolution 3D *T*_1_-weighted MPRAGE image was acquired for each participant (*FOV* = 256 × 256 × 160 mm; *TR* = 8.5 ms; *TE* = 3.9 ms; acquisition time = 7.3 min; voxel size = 1 × 1 × 1 mm). One hundred and fifty resting state (eyes shut) functional scans were acquired using a using a T^*^_2_ weighted gradient echo sequence to visualize changes in the BOLD signal (*TR* = 2000 ms, *TE* = 28 ms; flip angle = 90°; *FOV* = 240 × 240 mm; voxel size: 3 × 3 × 3.5 mm, slice gap 0.35 mm; 38 slices; acquisition time = 5.06 min). Diffusion weighted data were encoded along 32 independent directions, with one non-diffusion weighted image, using a single-shot echo-planar imaging (EPI) sequence with SENSE parallel imaging scheme (SENSivitiy Encoding; *TR* = 12052 ms; *TE* = 55 ms; *B*-value 1000; slice thickness/gap FOV; slice number = 70; voxel dimensions 2 × 2 × 2 mm; acquisition time 8.08 min).

### Statistical analysis of behavioral data

Behavioral data were analysed using SPSSv16. Two sample *t*-tests were used to examine group differences in age and IQ measures. Correlations were performed to examine relationships between structural and functional connectivity, between connectivity values and ADI-R scores and between connectivity values and striatal activation to social rewards. For correlations with the ADI-R, the DSM-5 model, which classifies ASD symptoms into social and communicative deficits (SCD) and restricted and repetitive behaviors (RRB), was used. The two factor model has been supported by a number of factor analytic studies (Boomsma et al., 2008; Frazier et al., 2008; Georgiades et al., 2012; Mandy et al., 2012). Item level data were classified into SCD or RRB symptom domains according to the two factor model reported by Georgiades et al. (2012) to create a quantitative score on each factor. Pearsons's and Spearman's rank-order correlations were used where appropriate.

### Functional connectivity analysis

**Figure 1:**
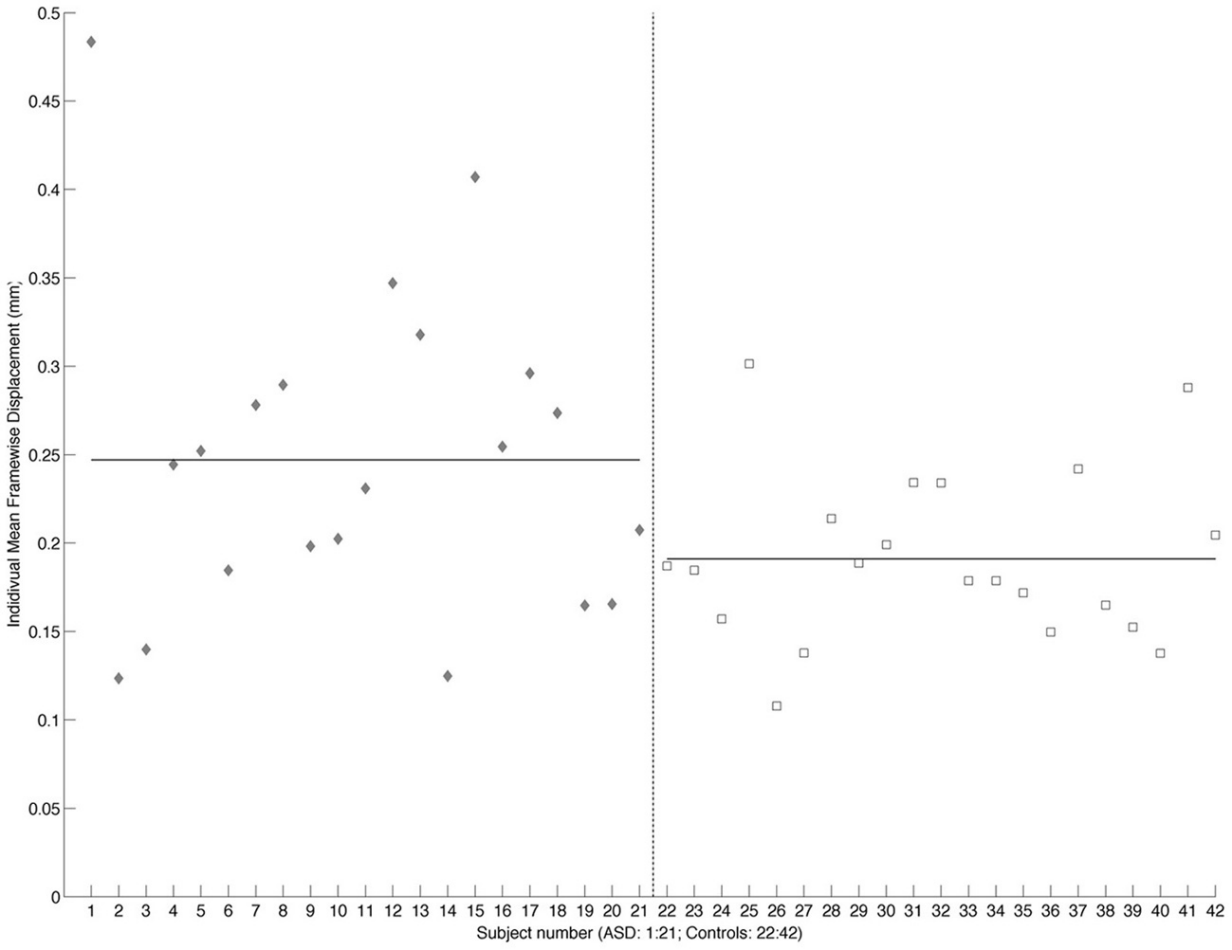
**Scatterplot showing individual mean framewise displacement.** Individual subjects are shown on the *x*-axis and framewise displacement (mm) on the *y*-axis. ASD subjects are shown as gray diamonds and controls as white squares. The dashed vertical line divides the two groups. Solid horizontal lines across the ASD and Control values show the group mean.

fMRI preprocessing was carried out in SPM8 (www.fil.ion.ucl.ac.uk/spm) in Matlab, 2009a (MathWorks Inc., United Kingdom). Before preprocessing, the origin was set to the anterior commisure for both *T*_1_-weighted and EPI Images. The images were slice-time corrected, realigned to correct for motion artifacts and co-registered to the skull stripped *T*_1_-weighted image. Normalization to standard stereotaxic space (Montreal Neurological Institute; MNI) was performed using the ICBM EPI template and the unified segmentation approach (Ashburner and Friston, 2005). The data were then re-sliced to a voxel size of 2 × 2 × 2 mm^3^. Finally, the images were smoothed using a 5 mm full-width-half-maximum (FWHM) Gaussian kernel to conform to assumptions of statistical inference using Gaussian Random Field Theory (Friston et al., 1995a,b). Given recent evidence that resting-state networks are particularly susceptible to head motion (Power et al., 2012; Van Dijk et al., 2012) independent samples *t*-tests were performed to ensure that groups did not differ on rotation or translation parameters [translation: mean *ASD* = 0.0401 (*SD* = 0.016), mean control = 0.0331 (*SD* = 0.0157) *p* = 0.136; rotation: mean ASD = 0.0006 (*SD* = 0.00002), mean control = 0.0005 (*SD* = 0.00002) *p* = 0.122] and average frame-wise displacements (see Figure 1) were included as covariates of no interest in the analyses as findings from a recent resting-state study indicate that this yields similar results to removing high-movement time-points (scrubbing) (Fair et al., 2012; Di Martino et al., 2013; Satterthwaite et al., 2013; Yan et al., 2013).

**Figure 2:**
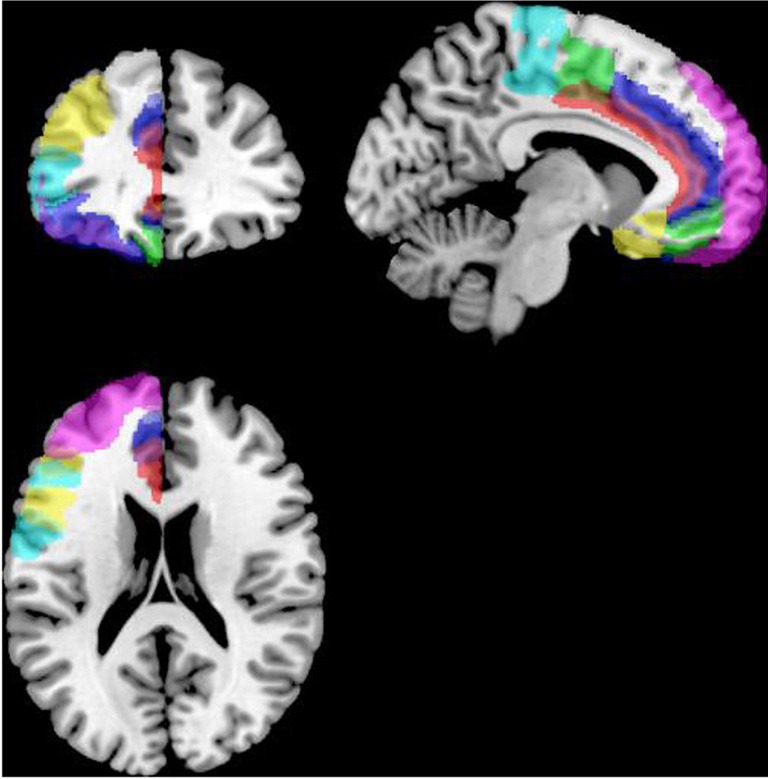
**Masks for the frontal cortex (only the left hemisphere is shown).** ACC is shown in red, the OFC in blue, the MPFC in green, frontal pole in violet, IFG opercularis in yellow, IFG triangularis in cyan, juxapositional lobe in green, MFG in yellow, paracingulate in blue, precentral gyrus in light blue, SFG in grayscale and the subcallosal gyrus in yellow, displayed on the left hemisphere of a standard brain in neurological convention (left is left and right is right).

**Figure 3:**
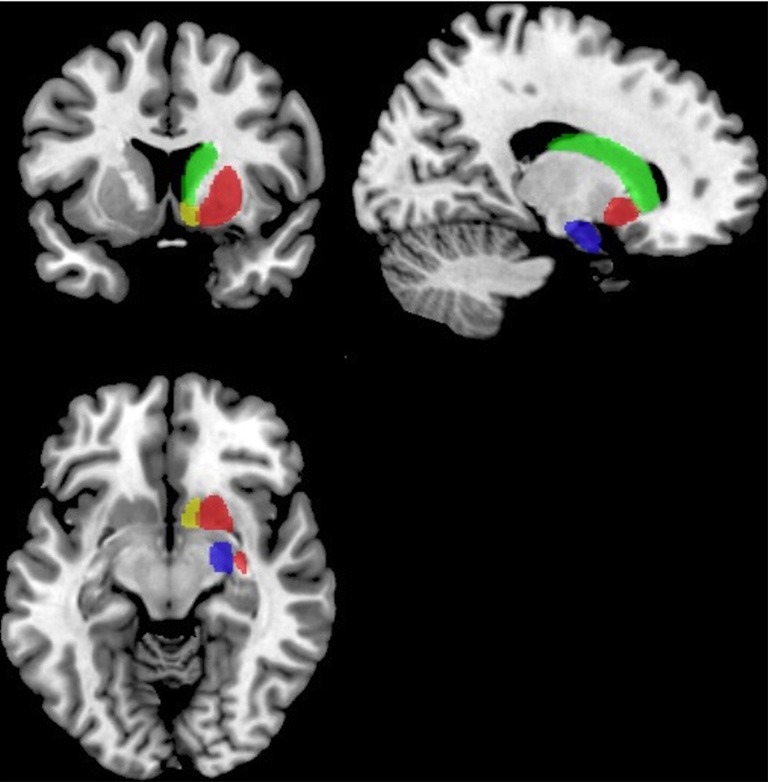
**Masks for the striatum and amygdala.** The NAcc is shown in yellow, the caudate in green, the putamen in red and the amydgala in blue displayed on the right hemisphere of a standard brain in neurological convention (left is left).

Functional connectivity analysis was carried out using the CONN toolbox (http://www.nitrc.org/projects/conn/) (Whitfield-Gabrieli and Nieto-Castanon, 2012). Normalized bias corrected *T*_1_ images were generated in SPM (http://www.fil.ion.ucl.ac.uk/spm/) and segmented into gray matter, white matter, and CSF. The principle eigenvariate of the BOLD time-courses from white matter and CSF, as well as the 6 motion correction parameters were included as regressors in the analysis to remove signals associated with these factors. The data were then band pass filtered between 0.008 and 0.2 Hz as recommended by Baria et al. (2011). A hanning window was used to weight down the initial and end scans within the resting state period. Seed regions were defined within the left and right frontal cortex [including the frontal medial and orbital cortices, inferior frontal gyrus, pars opercularis and pars triangularis, frontal pole, middle, superior frontal gyrus, subcallosal cortex, cingulate gyrus-anterior division, the paracingulate gyrus, precentral gyrus, and juxtapositional lobule cortex/supplementary motor area (see Figure 2)]. As the amygdala provides important inputs to the striatum (Haber, 2003; Groenewegen et al., 2003; Haber and Knutson, 2009) and has been implicated in functional and structural MRI studies of ASD (Baron-Cohen et al., 2000; Schultz, 2005; Verhoeven et al., 2009; Groen et al., 2010; Greimel et al., 2012a,b; Sato et al., 2012), it was also included as a seed region in this analysis (see Figure 3). Target regions included the left and right caudate, putamen, and NAcc (see Figure 3). Masks for these regions were generated using the Harvard-Oxford probabilistic atlas in FSL (http://fsl.fmrib.ox.ac.uk/fsl/fslwiki/) and thresholded at 20%. The ROI time series were defined as the principle eigenvariate of the time series within the ROI voxels using principle component decomposition. ROI-to-ROI correlational analyses were performed between each of the seed regions in the frontal cortex and amygdala and the target regions in the striatum. Second level random effects analyses were computed to examine group differences in connectivity using a *t*-test with age, IQ, and frame-wise displacements included as covariates to control for the effects of these factors. Results were corrected for multiple comparisons for the target regions at the FDR threshold (*p* < 0.05).

### Diffusion tensor tractography

Preprocessing of diffusion weighted data was carried out using Explore DTI (Leemans et al., 2009). The data were first screened by looping through each subjects' image to ensure that there were no gross artifacts such as signal dropout. Data were then corrected for eddy current distortions and subject motion with b-matrix rotation to preserve orientational information (Leemans and Jones, 2009). First, the diffusion-weighted images were realigned to the non-diffusion weighed (B0) image using a full affine transformation and cubic interpolation. Motion tensor values were estimated using robust estimation of tensors by outlier rejection (RESTORE; Chang et al., 2005). The RESTORE method improves tensor estimation compared to the linear and non-linear least squares methods, correcting for distortions due to fat suppression and cardiac pulsation. The final preprocessing step involved correcting for physically implausible signals. The data were then visually inspected to ensure that the gradient components were in the correct orientation. Finally, participants were excluded for excessive motion (>3 mm), with 22 ASD and 24 control participants retained for further analysis.

**Figure 4:**
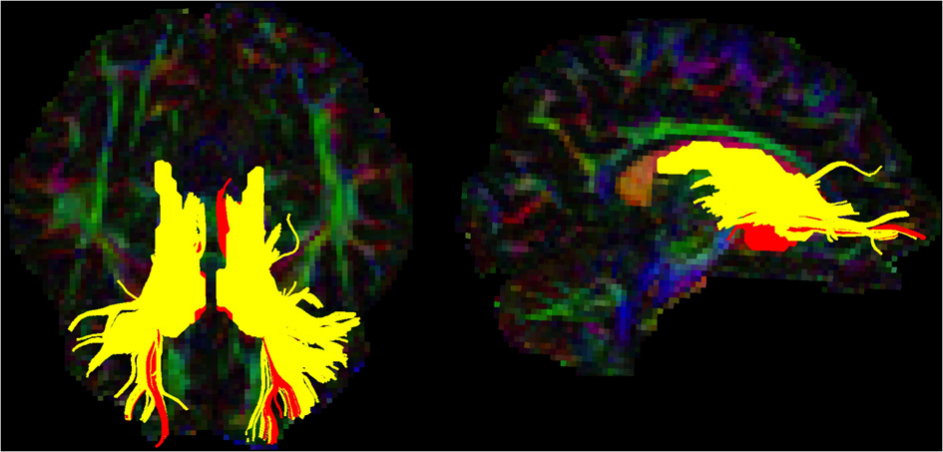
**Caudate and NAcc tracts for the template subject.** Tracts are shown in the axial (left) and sagittal (right) planes in neurological convention (left is left). The caudate-prefrontal tracts are shown in yellow and NAcc-prefrontal tracts are shown in red.

Tractography analyses were confined to intra-hemispheric tracts between regions that showed significant group differences in functional connectivity. Whole brain tractography was carried out using the deterministic streamline algorithm (Basser et al., 2000) as implemented in Explore DTI (Leemans et al., 2009). Tractography was carried out in each subjects' native space using a 2 mm seed point resolution, a 1 mm step size, an angle threshold of 30° and an FA tract termination threshold of 0.2. Specific tracts of interest were then isolated using regions of interest (ROIs) with inclusive Boolean logical “AND” operators used to include tracts passing through a specific regions and exclusion “NOT” operators used to exclude tracts passing through other regions. The atlas based segmentation approach was used to define ROIs in a template subject's native space (Lebel et al., 2008). These ROIs were then transformed to each subjects' native space for tractography analysis. A template subject was chosen at random as in Lebel et al. (2008). Masks of the caudate and NAcc from the Harvard-Oxford atlas, and a mask of the frontal cortex from the MNI atlas were created in FSL and thresholded at 20% in SPM8. These masks were then transformed to the template subjects native space by (i) co-registering the subjects T1 image to the subject's motion distortion corrected FA map (ii) multiplying the masks by the inverse transform parameters (MNI->Native space) generated using the segmentation option in SPM, (iii) re-slicing the masks to the same dimensions as the FA map and binarising them using the “imcalc” option in SPM. These masks were then visually inspected to ensure that they provided a good fit to the anatomical structure. Tractography analysis was carried out in the template subject using these inclusion masks (see Figure 4). “AND” gates were then placed at the caudate and NAcc to include only the regions from which tracts projected to the PFC. NOT gates were drawn in the planes across the midline and the posterior commisure, and to exclude motor tracts, cortico-spinal tracts, tracts from the corpus callosum and tracts to the temporal lobe. The atlas based segmentation tool was used to carry out tractography analysis in each subject's native space using the ROIs transformed into the subject specific space for each tract as this method has been successfully applied to improve tract delineation (Verhoeven et al., 2010). An upper limit of 100 mm was placed on the tract length. Outliers were excluded for each group separately for FA, MD, RD and AD values that were greater than 1.5 box lengths from the inter-quartile range. Multivariate analyses were computed to compare groups in terms of FA, MD, RD, and AD.

## Results

Groups did not differ in terms of age or IQ (see Table 1).

**Table 1:** **Mean age and IQ scores**.

	**Autism**	**Controls**	***P***
Age	17.28 (3.57)	17.15 (3.64)	0.545
**WASI**			
Full Scale IQ	109.25 (15.04)	111.85 (12.32)	0.889
Verbal IQ	108.54 (14.22)	110.52 (13.59)	0.967
Performance IQ	107.52 (14.68)	110.81 (11.11)	0.660

Standard deviations are shown in parenthesis.

### Striatal functional connectivity

#### Group-wise comparisons

**Figure 5:**
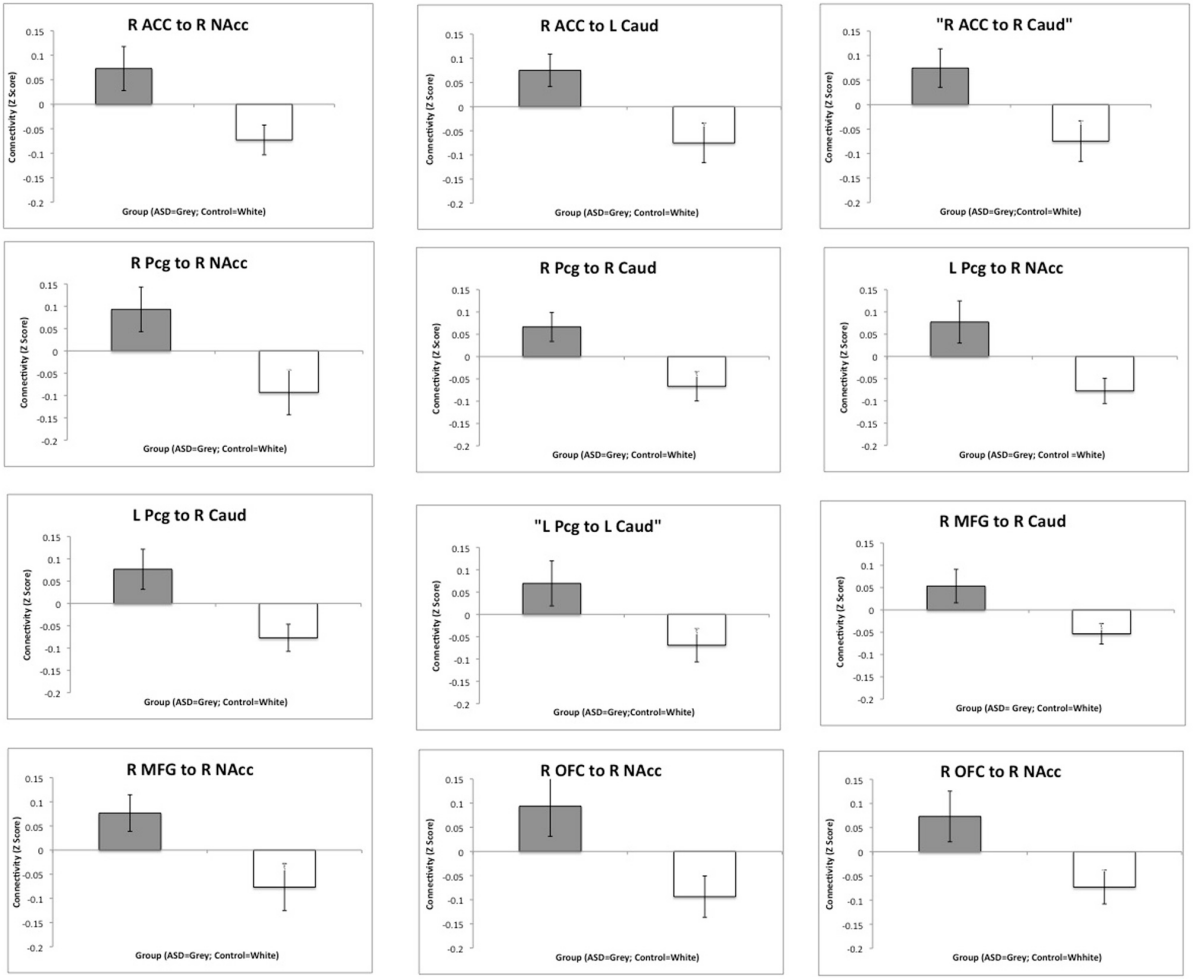
**Group differences in functional connectivity between the frontal cortex and the striatum.** Bar charts show Z-transformed *R*-Values for connectivity between each of the regions for which there was a significant group difference, adjusting for age, IQ and frame-wise displacements. The ASD group is shown in gray and the controls in white with standard error of the mean displayed. R, Right; L, Left; ACC, Anterior Cingulate Cortex; MFG, Middle Frontal Gyrus; Pcg, Paracingulate Gyrus; NAcc, Nucleus Accumbens; Caud, Caudate.

Regions showing significantly increased functional connectivity between the frontal cortex and the striatum in the ASD group are listed in Table 2. There were no regions that showed significantly reduced connectivity between the frontal cortex and the striatum and there were no significant group differences in connectivity between the amygdala and striatum. Bar charts showing z-transformed *r*-values, adjusted for age, IQ and frame-wise displacements, for connectivity between each of the regions for which there was a significant group difference can be seen in Figure 5. The ASD group showed significant positive connectivity between regions for which there were significant connectivity differences between groups, whereas controls showed negative connectivity between these regions at rest, when adjusting for age, IQ and frame-wise displacements. With the exception of right MFG to NAcc connectivity, negative connectivity was no longer apparent between frontostriatal regions in controls when covariates were not included in the analysis. Within group values for regions showing significant group differences in connectivity can be seen in Table 3.

**Table 2:** **T-scores and *p*-values for regions showing significantly increased connectivity in the ASD group, controlling for age, IQ and frame-wise displacements**.

**Source**	**Target**	***T*-Value**	***P*-Unc**	***P*-FDR**
Right cingulate gyrus, anterior division	Right accumbens	2.52	0.016	0.032
	Right caudate	2.72	0.009	0.029
	Left caudate	2.98	0.005	0.029
Right middle frontal gyrus	Right accumbens	2.68	0.011	0.044
	Right caudate	2.56	0.015	0.044
Right paracingulate gyrus	Right accumbens	3.24	0.003	0.014
	Right caudate	3.01	0.005	0.014
Right frontal orbital gyrus	Right accumbens	2.67	0.011	0.048
	Left accumbens	2.53	0.016	0.048
Left paracingulate gyrus	Right accumbens	2.92	0.006	0.018
	Right caudate	3.04	0.004	0.018
	Left caudate	2.38	0.023	0.045

**Table 3:** **Within-groups t-scores and p-values for regions showing group differences in functional connectivity controlling for age, IQ and frame-wise displacements**.

**Source**	**Target**	***T*-value**	**P-FDR**
Right cingulate gyrus, anterior division	Right accumbens	ASD = 2.53	ASD = 0.0314
		Control = −2.35	Control = −0.0486
	Left caudate	ASD = 2.98	ASD = 0.0299
		Control = −2.76	Control = 0.0486
	Right caudate	ASD = 2.67	ASD = 0.0314
		Control = −2.36	Control = 0.049
Right middle frontal gyrus	Right accumbens	ASD = 2.73	ASD = 0.0378
		Control = −2.68	Control = 0.0355
	Right caudate	ASD = 2.62	ASD = 0.0378
		Control = −2.64	Control = 0.0355
Right paracingulate gyrus	Right accumbens	ASD = 3.29	ASD = 0.0118
		Control = −3.55	Control = 0.0006
	Right caudate	ASD = 3.07	ASD = 0.0118
		Control = −3.14	Control = 0.0009
Right frontal orbital gyrus	Right accumbens	ASD = 2.67	ASD = 0.0484
		Control = −2.47	Control = 0.07
	Left accumbens	ASD = 2.38	ASD = 0.0484
		Control = −1.91	Control = 0.09
Left paracingulate gyrus	Right accumbens	ASD = 2.98	ASD = 0.0149
		Control = −3.05	Control = 0.0247
	Right caudate	ASD = 0.3.00	ASD = 0.0149
		Control = −2.68	Control = 0.0328
	Left caudate	ASD = 2.39	ASD = 0.0436
		Control = −2.25	Control = 0.06

#### Correlations with social reward processing

**Figure 6:**
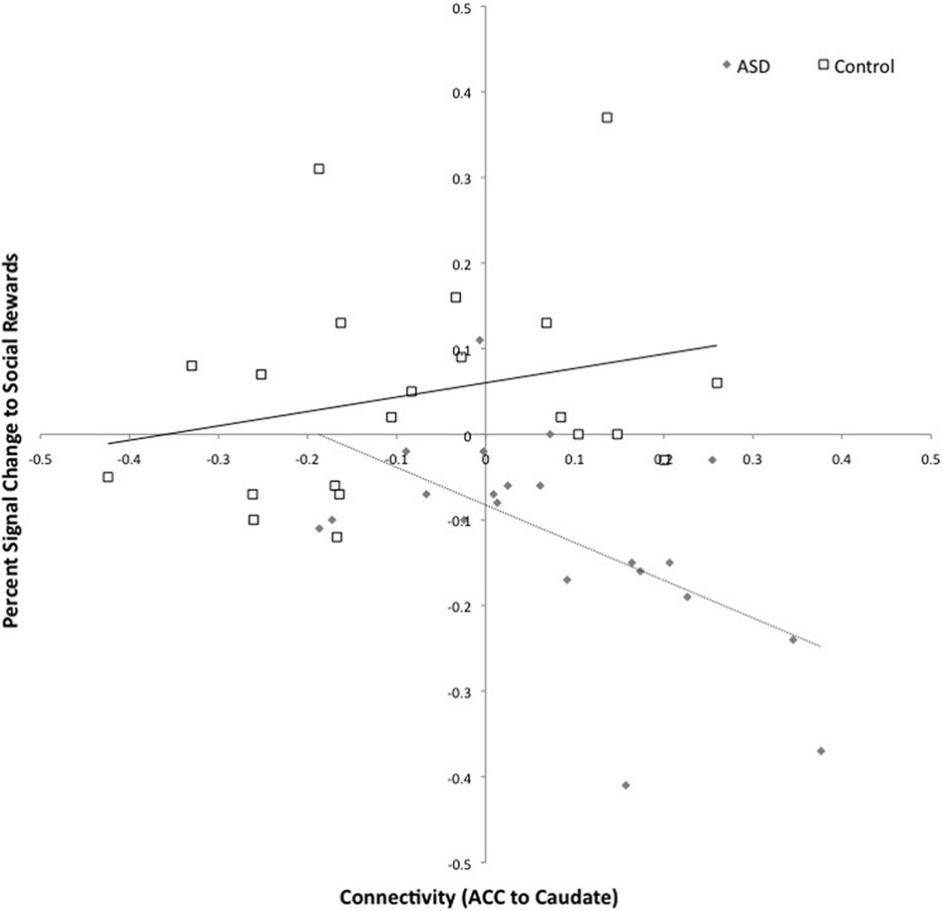
**Connectivity between the left caudate and right anterior cingulate and activation to social rewards in the left caudate.** Connectivity values are shown on the *x*-axis and percent signal change for social reward feedback is shown on the *y*-axis. The ASD group is shown in gray (with dashed trend-line) and the controls in white (with solid black trend-line).

The same participants previously completed an fMRI study of social and monetary reward processing (Delmonte et al., 2012), the results of which indicated that the ASD group showed deactivation to social rewards in the left caudate. We therefore explored whether increased connectivity between the right ACC and the left caudate in ASD was associated with deactivation to social rewards. There was a negative correlation between connectivity and SID activation in ASD but not controls (*ASD*: *r* = −0.576, *p* = 0.006; CON: *r* = 0.234; *p* = 0.307), see Figure 6. In the ASD group, deactivation to social rewards in the left caudate was associated with increased connectivity between the left caudate and the anterior cingulate.

#### Correlations with behavior

**Figure 7:**
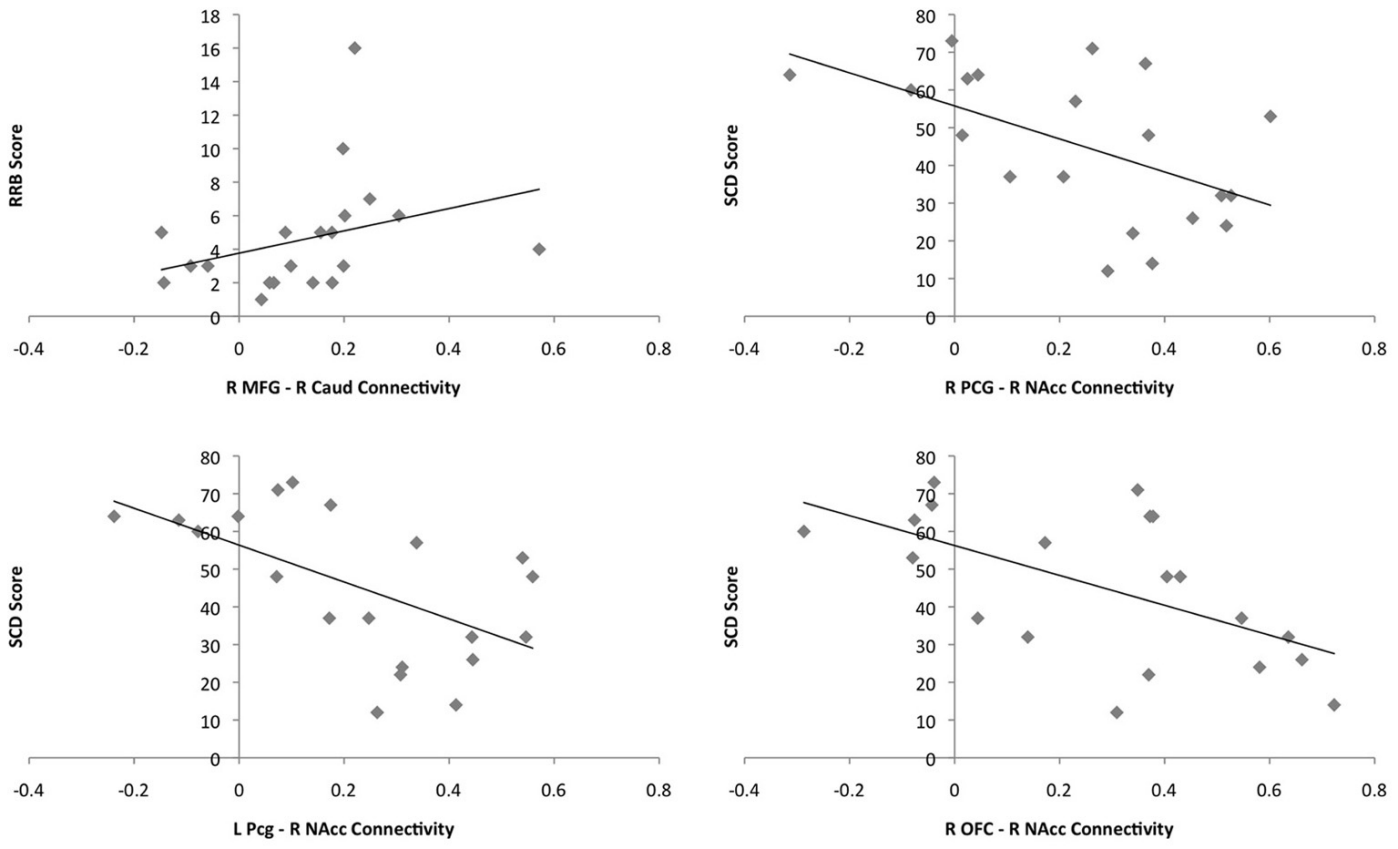
**Plots of the correlations between connectivity values and behavioral measures for the ASD group.** Connectivity values are shown on the x-axes and scores on the behavioral measures on the y-axes. ASD single subjects are represented as gray diamonds (solid black trend-line).

There was a positive correlation between connectivity in the right MFG and the right caudate and RRB in the ASD group (*r* = 0.573, *p* = 0.008); greater connectivity was associated with greater impairment. Connectivity between the right and left Pcg and the right NAcc was negatively correlated with SCD in the ASD group (*r* = −0.511, *p* = 0.012; *r* = −0.572; *p* = 0.008); greater connectivity was associated with less impairment. Similarly, there was a negative correlation between connectivity between the right OFC and right NAcc and SCD score in the ASD group (*r* = −0.519; *p* = 0.019). Associations between connectivity values and behavioral measures can be seen in Figure 7. These correlations did not withstand correction for multiple comparisons at the bonferroni level [*p*_(0.05/24)_ = 0.002]. Twenty-four correlations were performed as there were twelve regions showing significant group differences in functional connectivity and 2 behavioral measures. Figure 7 shows plots of the correlations between connectivity values and behavioral measures in the ASD group.

### Striatal structural connectivity

Multivariate analyses with age, I.Q. and TIV entered as covariates indicated that there were no significant between group differences in FA, MD, RD, or AD in the tracts of interest.

### Correlations between structural and functional connectivity

There was a significant positive correlation between AD in the right caudate to prefrontal tract and functional connectivity (raw z-scores) between the right MFG and the right caudate across the group as a whole (*r* = 0.414, *p* = 0.010), however, a within group analysis showed only a trend in the ASD group (*r* = 0.445, *p* = 0.056) and no relationship with control participants (*r* = 0.214, *p* = 0.380) indicating that the significant correlation was largely driven by variance in the ASD group. There were no other significant correlations between functional and structural connectivity.

## Discussion

The ASD group showed increased functional connectivity between the ACC, Pcg, OFC, and the MFG in the prefrontal cortex and the caudate and NAcc in the striatum, with group differences primarily in the right hemisphere. Increased functional connectivity between frontostriatal regions in ASD was associated BOLD deactivation to social rewards (Delmonte et al., 2012) and behavioral measures of SCD and RRB. There were no significant group differences in the structure of frontostriatal tracts. This suggests that group differences in functional connectivity, reported in the present study, may not be due to alterations in frontostriatal structural connectivity in ASD, though these findings could also reflect methodological issues associated with DTI tractography.

### Group differences in functional connectivity

#### Hyperconnectivity between the anterior cingulate cortex (ACC) and striatum in ASD

Neuranatomical connections between the ACC and the striatum are organized in functionally distinct loops. The ventral ACC is connected to the ventral and dorsal striatum (VS and DS) and the dorsal ACC to the DS (Beckmann et al., 2009). ACC regions connected to the VS are involved in emotion, reward and pain whereas regions connected to the DS are mostly involved in motor functions, conflict/error detection and reward (Beckmann et al., 2009). The dorsal cognitive division of the ACC is connected to other regions involved in attention including the dorsolateral prefrontal cortex (dlPFC) and parietal attention regions. The rostral-ventral affective division is connected to limbic regions including the OFC, amygdala, and periaqueductal gray (PAG) (Bush et al., 2000).

Previous findings, together with the present results, suggest that hyperconnectivity between the ACC and caudate may be specific to adolescents/adults with ASD. Increased bilateral connectivity between the ACC and caudate has been reported during visuomotor performance among adults with ASD (Turner et al., 2006) but not resting state among children with ASD (Di Martino et al., 2010). ACC pathology has also been implicated more generally in functional and structural neuroimaging studies of ASD. In a meta-analysis of functional neuroimaging studies, hypoactivation was reported in the perigenual ACC in ASD during social tasks and in the dorsal ACC for non-social tasks (Di Martino et al., 2009). Reduced ACC gray matter volume (Haznedar et al., 2000; Greimel et al., 2012a,b) and surface area (Hadjikhani et al., 2006; Doyle-Thomas et al., 2012), primarily in the right hemisphere, have also been reported.

Hyperconnectivity between the right ACC and the left caudate was associated with deactivation to social rewards in ASD as reported in a previous study among the same participants (Delmonte et al., 2012). This is in keeping with the role of the ACC in social perception and social cognition deficits in ASD (Di Martino et al., 2009) and with recent evidence of abnormal ACC activation during social and non-social reward processing (Dichter et al., 2011; Kohls et al., 2012a,b)—although we did not observe the latter in our previous study. Taken together these results suggest that abnormal activation in the left caudate during social reward feedback may have been due to abnormal top–down processes governed by the ACC.

#### Hyperconnectivity between the paracingulate (Pcg) and striatum in ASD

The Pcg is often thought of as part of the ACC (Gallagher and Frith, 2003; Walter et al., 2005), though it is anatomically, and perhaps functionally, distinct from the ACC (Gallagher and Frith, 2003). Diffusion MRI data in humans indicates that it is connected to the VS and DS and the dorsal prefrontal cortex (Beckmann et al., 2009). The Pcg is involved in emotion, social interaction, reward and decision-making, conflict monitoring and error detection (Vogt, 2005; Amodio and Frith, 2006; Beckmann et al., 2009). The anterior Pcg, along with the superior temporal sulci and the temporal poles, plays an important role in theory of mind (Gallagher and Frith, 2003; Walter et al., 2005) with activation modulated by the amount of social interaction involved in the task (Walter et al., 2004). The Pcg and striatum are thought to be involved in separate phases of decision-making, with the Pcg involved in action selection and the VS responding to positive outcomes (Rogers et al., 2004).

Previous functional connectivity studies of the striatum in ASD have not implicated the Pcg (Turner et al., 2006; Di Martino et al., 2010), however, reduced connectivity between the Pcg and the intraparietal sulcus during working memory task performance (Koshino et al., 2005) and reduced connectivity with the IFG during sentence comprehension have been reported in ASD (Just et al., 2004). Additionally, reduced Pcg activation during theory of mind tasks (Kana et al., 2009) and reduced gray matter volume in the right Pcg (Abell et al., 1999) have been reported. In the present study increased connectivity between the Pcg and the NAcc was negatively associated with SCD deficits, suggesting that increased connectivity between these regions in people with high functioning ASD could reflect a compensatory mechanism.

#### Hyperconnectivity between the middle frontal gyrus (MFG) and striatum in ASD

The MFG, along with part of the SFG, comprises the dlPFC (Barbas and Pandya, 1989; Badre and D'Esposito, 2009; Yeterian et al., 2012), which is connected to the rostral dorsolateral caudate as well as the OFC and medial prefrontal cortex (mPFC) (Haber, 2003; Lehéricy et al., 2004; Leh et al., 2007; Draganski et al., 2008). The dlPFC is involved in a host of executive functions including working memory, set-shifting, rule learning, and planning (Goldman-Rakic et al., 1996; Leung et al., 2002; Badre and D'Esposito, 2009) and is thought to work together with the caudate to mediate these functions (Haber, 2003; Pasupathy and Miller, 2005). In terms of rule-learning, rewarded associations are thought to be identified in the striatum, which trains slower learning mechanisms in the dlPFC (Pasupathy and Miller, 2005). The dlPFC is involved in rule-learning via reinforcement; once the rule has been acquired, the dlPFC is no longer required and action execution is controlled by the premotor cortex (Badre and D'Esposito, 2009).

As in previous studies of striatal connectivity (Turner et al., 2006; Di Martino et al., 2010), there was a significant increase in connectivity between the caudate and MFG in ASD. In addition, the ASD group showed hyperconnectivity between the MFG and the NAcc. This is in keeping with a body of evidence implicating the MFG/dlPFC in ASD. Decreased functional connectivity has been reported between the dlPFC and the visuospatial regions in the occipital and parietal lobes during visuospatial processing (Damarla et al., 2010). ASD subjects also show less negative correlation between the dlPFC and amygdala during passive viewing of emotional facial expressions (Rudie et al., 2011) and increased regional homogeneity (local synchronization of the BOLD signal) in the right MFG during rest (Paakki et al., 2010). Reduced activation in the dlPFC during social and non-social information processing, including spatial working memory (Luna et al., 2002), sustained attention (Christakou et al., 2012) and memory encoding of social information have been recorded (Greimel et al., 2012a,b) as well as abnormal involvement in tasks such as gaze perception (Vaidya et al., 2011). In addition, increased gray matter volume (Ecker et al., 2012) and neuronal number (Courchesne et al., 2011) indicate structural abnormalities in the dlPFC in ASD.

Connectivity between the right MFG and right caudate was associated with increased RRB. This in keeping with previous literature implicating the frontostriatal circuitry, particularly the caudate and MFG/dlPFC, in executive function and repetitive behavior deficits in ASD (Hollander et al., 2005; Rojas et al., 2006; Estes et al., 2011; Langen et al., 2011a; Ecker et al., 2012) and suggests that cognitive as opposed to sensorimotor circuitry is implicated in repetitive behaviors in high functioning ASD.

#### Hyperconnectivity between the orbitofrontal cortex (OFC) and striatum in ASD

The OFC is involved emotion, motivation and reward, and is the region of prefrontal cortex most often associated with reward-guided decision-making, subserving both sensory and abstract reward processing (Haber, 2003; Haber and Knutson, 2009; Rushworth et al., 2011). Specifically, OFC activity is thought to reflect signal valuation, for both rewards and punishments, tracking expected reward value prior to decision-making and the received reward value after a choice has been made (Rushworth et al., 2011). Efferent connections from the OFC provide input to the VS, with the VS also receiving input from the amygdala and hypothalamus (Haber, 2003; Draganski et al., 2008). The OFC, together with the VS and amygdala, is thought to compute the salience value of social stimuli, with this circuitry playing a potential role in social motivation deficits in ASD (Chevallier et al., 2012; Kohls et al., 2012a,b).

Previous fMRI studies have indicated abnormal activation of the OFC, VS and amygdala during both social and non-social reward processing in ASD (Scott-Van Zeeland et al., 2010; Dichter et al., 2011, 2012; Kohls et al., 2012a,b), providing support for the hypothesized role of these regions in social motivation difficulties in ASD. Additionally, structural alterations have been recorded in the OFC in ASD, including decreased gray matter volume (Ecker et al., 2012), increased cortical thickness (Hyde et al., 2010) and altered sulcogyral morphology (Watanabe et al., 2013). Previous examinations of frontostriatal functional connectivity in ASD have not specifically implicated abnormal OFC—VS connectivity (Turner et al., 2006; Di Martino et al., 2010). The results of the present study indicated that increased connectivity between the OFC and NAcc was associated with fewer SCD deficits, suggesting that increased connectivity between these regions may function to reduce social difficulties among adolescents/young adults with high-functioning ASD.

### Frontostriatal structural connectivity

There were no significant group differences in white matter microstructure (FA, MD, RD, AD) in tracts connecting the caudate or NAcc to the prefrontal cortex. Only one previous study has specifically examined microstructural integrity of frontostriatal circuits. Greater MD was reported in projections between the right NAcc and prefrontal cortex but not in projections between the caudate and prefrontal cortex among adults with ASD (Langen et al., 2011a,b). The disparity between the present findings and those of Langen et al. (2011a,b) could be due to age differences between the samples, with the sample in the present study being younger than those previously examined. The difference between structural and functional connectivity findings in the present study, with significant group differences for functional data but not structural data, may be due to several factors. Resting state connectivity analysis is not anatomically constrained therefore differences in connections between the striatum and PFC could potentially arise from structural alterations in another part of the circuit, for example in fiber pathways connecting the striatum and pallidum, pallidum and thalamus, or thalamus and cortex. Frontostriatal connections may be characterized by topographical reorganization of fiber pathways in ASD rather than microstructural alterations. This could be explored using connectivity based classification methods (Behrens et al., 2007). Another potential explanation is that structural data may be less sensitive to group differences than functional data (Finger et al., 2012) or that subtle white matter differences may remain undetected by the typical “tract averaged” approach used in most tractography studies and may require the use of “tract resampling” techniques to capture more subtle variations over the length of a tract (Colby et al., 2012). Finally, with the exception of a significant correlation between functional connectivity between the right caudate and MFG, and AD in the right caudate to prefrontal tract, measures of functional connectivity were unrelated to structural metrics in the present study. Greater concordance between functional and structural connectivity metrics may be obtained by examining specific loops (i.e., cingulo-striatal loops or dlPFC-striatal loops) in frontostriatal circuitry rather than connections between the striatum and the entire frontal cortex. It is likely that such analyses would require high-resolution diffusion imaging (HARDI) data and advanced modeling techniques such as constrained spherical deconvolution (CSD) rather than the tensor model used here.

### Limitations and future directions

The results of the present study should be interpreted in the light of several methodological issues. We did not replicate previous findings showing positive functional connectivity between frontostriatal regions, for example between the MFG and the caudate, in our control group (Di Martino et al., 2008). This is perhaps due to developmental factors related to the age range of the participants in the present study. Indeed negative connectivity between frontostriatal regions in controls was no longer apparent when covariates were not included in the analyses. Another potential explanation is that Di Martino et al. (2008) divided the caudate into ventral and dorsal regions, which showed distinct patterns of connectivity with sub-regions of the ACC and dlPFC, whereas we examined connectivity using gross morphological boundaries. Examining connectivity across entire structures in the current study may have obscured functional relationships between sub-regions of these structures. This can be circumvented to some extent by using a seed-to-voxel approach rather than the ROI-to-ROI approach taken in this study. However, the seed-to-voxel approach also requires a significantly greater number of statistical comparisons, which can potentially lead to Type II errors (false negatives). Given that ASD is a functionally heterogeneous population and this study has a relatively small sample size (*N* = 21), the ROI-to-ROI approach used in the present study is likely to have been more sensitive to group differences. Recent studies have shed light on the topography of functional and structural connections within the striatum (Robinson et al., 2012; Verstynen et al., 2012; Tziortzi et al., 2013) which may be useful in defining seed regions for future studies of functional and anatomical connections in frontostriatal circuitry in ASD.

A limitation of functional connectivity methods used in the present study is that one cannot infer the source of differences in functional connectivity. Frontostriatal loops are part of larger circuitry which also involve thalamo-cortical connections (Haber and Knutson, 2009). Increased connectivity between the thalamus and frontal cortical regions has been reported in ASD (Mizuno et al., 2006), indicating that thalamo-cortical circuitry is also abnormal in ASD, which could impact on frontostriatal circuitry. Given the looped structure of cortico-striatal-thalamo-cortical connections (Alexander et al., 1986, 1990), and various regulatory influences on this circuitry (Haber and Knutson, 2009), it is difficult to infer at what point dysregulation occurs, i.e., in the frontal cortex, the thalamus, the striatum, other regulatory subcortical structures, or in specific connections between these structures. We did not examine the connectivity of the midbrain—which provides important dopaminergic input to the striatum (Schultz et al., 1997; Haber and Knutson, 2009)—due to the fact that the midbrain is particularly susceptible to artifacts from cardiac (Greitz et al., 1992; Dagli et al., 1999) and respiratory (Raj et al., 2001) signals. Future studies could examine midbrain function in ASD using optimized fMRI methods (Limbrick-Oldfield et al., 2012), could include additional ROIs in regions such as the midbrain and thalamus, and could use effective connectivity modeling techniques to more fully characterize connectivity within frontostriatal circuitry (and potentially shed light on the source of hyperconnectivity in this circuit) in ASD.

The lack of group differences in structural connectivity should be interpreted in the light of several factors. Firstly, DTI is associated with a number of confounds (Jones, 2010). The tensor model cannot characterize diffusion in regions of complex fiber architecture, or “crossing fibers” where fibers kiss, twist, splay kink, or bend (Basser et al., 2000; Frank, 2001; Tuch, 2004; Jones, 2010). This is an important issue given that crossing fibers are thought to make up to 90% of white matter (Jeurissen et al., 2012). Tensor derived metrics are also influenced by acquisition parameters, such as the *b*-value (Vos et al., 2012), which may further confound results. Improved understanding of brain structural connectivity in ASD will therefore require the use of HARDI methods such as CSD tractography. Another potential concern is that the presence of subtle differences along white matter fiber tract may remain undetected as the diffusion metrics are typically averaged along the entire tract segment under investiagtion, thus masking subtle and highly localized regions of effect. Emerging tractography techniques that assess variations in the diffuison meteric along the tract using a “tract resampling mechanism” have been shown to potentially increase the sensitivity of analyses to the presence of very subtle but important white matter fiber differences (Colby et al., 2012). Again the use of HARDI methods may provide futher insight into more subtle stuctural differences in ASD. Another potential methodological issue is that the age range of the participants in the present study may have introduced heterogeneity in the data due to ongoing developmental processes, which could have reduced power to detect group differences. Previous studies suggest that both gray and white matter undergo different developmental trajectories in ASD (Carper et al., 2002; Keller et al., 2007; Langen et al., 2009; Cheng et al., 2010; Mak-Fan et al., 2012), therefore future studies should use tighter age ranges to limit heterogeneity for group-wise comparisons. Finally, the size of the sample in the present study may have reduced power to detect potential group differences in structural connectivity.

Interestingly, hyperconnectivity between the PFC and the striatum was primarily lateralized to the right hemisphere in the present study. This is in keeping with evidence that differences in the structure and function of the ACC are largely lateralized to the right hemisphere (Haznedar et al., 2000; Bejjani et al., 2012; Dichter et al., 2012; Joshi et al., 2012), that increased gray and white matter volume asymmetries are lateralized to the right hemisphere (Herbert et al., 2005) and that regional homoegeneity, a measure of functional connectivity thought to index local synchrony in the BOLD signal, is primarily lateralized to the right hemisphere in ASD (Liu et al., 2008; Paakki et al., 2010). Future studies may wish to further examine potential hemispheric asymmetries in functional and structural connectivity in ASD.

## Conclusions

These results are in line with previous reports of increased functional connectivity between the striatum and frontal, temporal and parietal lobes as well as the pons in ASD (Turner et al., 2006; Di Martino et al., 2010). In the present study, hyperconnectivity was confined to limbic and associative frontostriatal circuits. Unlike previous studies (Di Martino et al., 2010), there were no group differences in sensorimotor loops. These findings add to a growing body of literature indicating significant increases as well as decreases in functional connectivity in ASD and do not support general under-connectivity accounts (Just et al., 2007), but suggest that ASD is characterized by complex functional re-organization which also involves hyperconnectivity within certain circuits. Increased functional connectivity in frontostriatal circuitry was associated with behavioral characteristics of ASD in terms of social interaction and communication and restricted interests/repetitive behaviors, as well as deactivation to social rewards in the striatum. There were no differences in structural connectivity as measured by DTI. This suggests that differences in functional connectivity were not detectable by DTI tractography in frontostriatal white matter but further research using advanced CSD based tractography is needed to clarify if subtle structural abnormalities exist in this region.

### Conflict of interest statement

The authors declare that the research was conducted in the absence of any commercial or financial relationships that could be construed as a potential conflict of interest.
